# Relationship between Gut microbiome and brain volumes among Japanese Men

**DOI:** 10.1371/journal.pone.0333612

**Published:** 2025-10-07

**Authors:** Sabrina Ahmed, Zhang Hexun, Yuichiro Yano, Yukiko Okami, Nazar Mohd Azahar, Keiko Kondo, Hisatomi Arima, Sayuki Torii, Mohammad Moniruzzaman, Gantsetseg Ganbaatar, Aya Kadota, Akira Andoh, Akihiko Shiino, Hirotsugu Ueshima, Katsuyuki Miura, for the SESSA Research Group

**Affiliations:** 1 NCD Epidemiology Research Center (NERC), Shiga University of Medical Science, Otsu, Shiga, Japan; 2 Department of Public Health, North South University, Dhaka, Bangladesh; 3 Department of Surgery, Shiga University of Medical Science, Otsu, Shiga, Japan; 4 Department of General Medicine, Juntendo University Faculty of Medicine, Tokyo, Japan; 5 Gunma University Center for Food Science and Wellness, Maebashi, Gunma, Japan; 6 Faculty of Health Sciences, Universiti Teknologi MARA, Cawangan Pulau Pinang, Kampus Bertam, Malaysia; 7 Department of Public Health and Preventive Medicine, Faculty of Medicine, Fukuoka University, Fukuoka, Japan; 8 Department of Internal Medicine, Shiga University of Medical Science, Otsu, Shiga, Japan; 9 Molecular Neuroscience Research Center, Shiga University of Medical Science, Otsu, Shiga, Japan; Nagoya University: Nagoya Daigaku, JAPAN

## Abstract

Evidence of preclinical interactions between the gut microbiome and brain health is accumulating. Studies of animal models and specific patient populations have suggested a relationship between gut microbiomes and brain volumes, but this association is understudied in apparently healthy humans. We conducted a population-based cross-sectional study of 623 Japanese men from the Shiga Epidemiological Study on Subclinical Atherosclerosis (SESSA). We performed 16S ribosomal RNA gene sequencing of stool samples collected during the follow-up stage (mean [SD] age, 68.0 [8.0] years; range, 46–83 years). All participants underwent brain magnetic resonance imaging and automated voxel-based morphometry. Principal coordinate analysis, linear discriminant, and multivariable linear regression analyses were performed. In multivariable linear regression analysis, after adjusting for age and total intracranial volume, only gray matter volume showed a positive association with alpha diversity (the Shannon index richness, q-value <0.01). However, no association was found after further adjustments for body mass index, physical activity, smoking, drinking, and hypertension. The weighted UniFrac distances (beta diversity) measured using principal coordinate analysis showed that lower and higher white and gray matter volumes formed distinct clusters (q < 0.01). In linear discriminant analysis and multivariable-adjusted linear regression analysis, several genera were significantly associated with gray and white matter volumes (q < 0.01); however, Lachnospiraceae, a butyrate-producing bacterium, was consistently related to a higher white matter volume in different statistical analysis models. Egarthellaceae, Bifidobacteraceae, and Selenomonadaceae showed a positive association with greater gray matter volume. Our findings support an association between gut microbiome diversity and brain volumes in middle-aged to older Japanese men. This study provides insight into the underlying effects of the gut microbiome on human brain volume.

## Introduction

The gut microbiota is a complex community of microorganisms living in humans’ and animals’ digestive tracts [1]. With the advancement of next-generation sequencing technology, elucidating the gut microbiome can facilitate outstanding contributions to the diagnosis, prevention, and treatment of various diseases [2]. Previous evidence has revealed that microbiota within the gut can greatly influence all aspects of physiology, including gut–brain communication, brain function, and even behavior [3]. Converging evidence suggests that the gut microbiota communicates with the central nervous system bidirectionally through the microbiome-gut-brain axis [4,5]. The microbiota secretes large amounts of brain-derived and neuronal trophic factors and short-chain fatty acids; these enzymes can cross the blood-brain barrier, affecting brain structure [6]. It is noteworthy that several brain disorders have been linked to imbalances in the microbial composition of the gut [7,8]. Prior studies have revealed age-related brain atrophy with a reduction in gray matter volume (GMV) in older adults with cognitive impairment [9,10]. Recent research suggests potential roles of the gut microbiota in patients with schizophrenia and depression, as changes in its composition are related to alterations in brain volumes (BVs) and function, particularly GMV and white matter volume (WMV) [11]. Preventing unhealthy aging through personalized or subpopulation-level microbiome-associated interventions in healthy humans is a new area of research [12,13]. We believe delaying or preventing age-dependent brain atrophy by maintaining gut flora is a crucial approach. Since there is no prior evidence of the association between the gut microbiome and BV in the general population, we aimed to assess this association in Japanese men.

## Materials and methods

### Study participants and design

The Shiga Epidemiological Study of Subclinical Atherosclerosis (SESSA) was a prospective observational study that examined the determinants of subclinical atherosclerosis in the Japanese population. The design of this study and information regarding baseline characteristics have been described previously [14,15]. In brief, from May 2006 to March 2008, investigators from SESSA randomly selected from the Basic Residents’ Register of Kusatsu City, Shiga Prefecture, by age strata and invited 2,379 Japanese men aged between 40 and 79 years. A total of 1,094 men participated in the baseline assessment (SESSA-1). Of them, 853 were reassessed at follow-up (SESSA-2). For this particular research, we used the data from the follow-up study (SESSA-2), where the recruitment of the study participants started on 1^st^ October 2010 and ended on 31st August 2014. Out of 853 men recruited in SESSA 2, 740 men went through SESSA MRI and voxel-based morphometry analysis; the remaining 113 participants were unable to join the analysis due to refusal. A total of 740 participants among the follow-up study participants underwent 1.5T magnetic resonance imaging (MRI) and voxel-based morphometry to measure brain structures, and 669 participants agreed to undergo the gut microbiology test during the follow-up. After excluding participants with a history of stroke (n = 44) and those with missing values for MRI or gut microbiology tests (n = 2), we finally included 623 participants for the current analysis (shown as a flow diagram in S1 Fig.). Written informed consent was obtained from all participants before they participated in the study. This study adhered to the code of ethics of the World Medical Association (1975 Declaration of Helsinki). This study was approved by the Institutional Review Board of Shiga University of Medical Science (G2008-61), and we adhere to the STROBE guideline (observational cross-sectional) to report the findings [16]. We have obtained written informed consent from each participant.

### BV and structures

The measurement of BV using MRI and voxel-based morphometry has been described in detail elsewhere [15,17]. High-resolution 3-dimensional T1-weighted spoiled gradient recalled brain MR images (repetition time = 13.5 ms, echo time = 5.8 ms, thickness = 1.6 mm/ − 0.8 mm, fractional anisotropy = 15°, frequency encoding = 288, 256 × 256 matrix) were obtained from participants at the follow-up (2012–2015) using 1.5-Tesla MR scanner (Signa HDxt 1.5 T; GE Healthcare, Milwaukee, WI) at the Shiga University of Medical Science Hospital. We used the automated morphometry toolbox of Statistical Parametric Mapping 12 with its extensions, such as Computational Anatomy Toolbox 12 software, to obtain BVs (global and six regions of interest [ROIs]) on a voxel-by-voxel basis. The details of the voxel-based morphometry approach have been mentioned elsewhere [18,19]. GMVs and WMVs were measured separately in milliliters and added together to obtain the total cerebral brain volume (TBV). Total intracranial volume (TIV) was quantified using automatic segmentation. We also quantified the BVs for a priori selected cognition-related ROIs, such as the hippocampus.

### Gut microbial measurements

Participants were provided with a brush-type fecal collection kit (TechnoSuruga Laboratory Co., Ltd., Shizuoka, Japan) containing preservation fluid that stably maintained the microbiota in feces in a broad range of temperatures (1–30°C) for one month. Stool fluid collected at a participant’s home was immediately returned to the Shiga University of Medical Science. DNA was extracted from fecal fluid using the bead-beating method [20] and subjected to 16S ribosomal RNA (16S rRNA) gene sequencing. DNA extracts were used as templates to amplify the V3-V4 region of each 16S rRNA gene with the primer pair 341F/805R using the two-step tailing polymerase chain reaction (PCR) method [21]. The paired-end sequencing of PCR amplicons was performed on the Illumina MiSeq platform (Illumina Inc., San Diego, CA, USA) at 2 × 300 base pairs. The details of this method have been described elsewhere [22].

### Preparing the microbiome database

Raw Illumina FASTQ files were acquired from the BaseSpace Sequence Hub (Illumina), and the sequences were analyzed using QIIME2 (version 2021.2) software. The quality of the joined sequences was filtered using the q2-demux plugin, followed by denoising with DADA2 to cluster and generate a feature table for analysis. The quality score of the raw data was visualized and removed from the point at which it started to decrease to below 30 for more than three consecutive reads on the forward and reverse sides. The taxonomic classification accuracy of the 16S rRNA gene sequences increased when a naive Bayes classifier was applied only to the ROI sequences. We created classifiers from the Silva 138 99% reference database by extracting the gene sequences necessary for this analysis and specifying the primers used for both the forward and reverse sequences.

### Assessment of covariates

Demographic data, medical history, medication use, and lifestyle factors, including smoking and alcohol consumption, were collected by trained research technicians using a self-administered questionnaire. Details of the assessment of covariates have been described elsewhere [14]. Participants reported their medication use history for hypertension, diabetes, and dyslipidemia. The history of smoking and alcohol consumption was also recorded. Smokers and alcohol drinking habits were defined as either “current” (smoking/consuming alcohol in the last 30 days), “past” (those who quit 30 days prior to the study), or “never” (those who never smoked/consumed alcohol). Body mass index (BMI) was defined as body weight (kg) divided by the square of the height (m). Body weight and height were measured while the participants wore light clothing without shoes. Blood pressure was measured using an automated sphygmomanometer (BP-8800SF; Omron Health Care, Kyoto, Japan) from the right arm with participants in a seated position after a strict 5-minute rest period. Hypertension was defined as systolic blood pressure of ≥140 mm Hg, diastolic blood pressure of ≥90 mm Hg, or use of antihypertensive medication. Participants who had exercised for more than 10 minutes or more over the previous 3 months were defined as physically active, and the data were recorded as the number of days per week of leisure-time physical activity [23].

After the measurement of blood pressure, venipuncture was performed early during the clinic visit, after at least a 12-hour fast. Samples were sent for routine laboratory tests, including the assessment of lipid profiles and glucose levels. Plasma glucose levels were measured from sodium fluoride-treated plasma using the hexokinase glucose-6-phosphate-dehydrogenase enzymatic assay. Glycated hemoglobin (HbA1c) was measured using the latex agglutination inhibition assay, according to either the Japan Diabetes Society (JDS) or National Glycohemoglobin Standardization Program (NGSP) protocol. JDS values were converted to NGSP values using the equation recommended by the JDS as follows: NGSP value (%) = 1.02 × JDS value (%) + 0.25 [24]. Diabetes mellitus was defined as fasting glucose of ≥126 mg/dL or HbA1c (NGSP) of ≥6.5%, or use of any antidiabetic medication.

### Statistical analyses

Descriptive statistics of participants are presented as percentages for categorical variables and as means (standard deviations, SDs) or medians (interquartile ranges, IQRs) for continuous variables. We controlled taxonomic analysis for multiple comparisons using the Benjamini-Hochberg method for false discovery rate (FDR) and measured the q value (FDR-adjusted P value). We performed linear regression to assess the independent association between alpha diversity and BV in terms of the Shannon index and richness according to an earlier study [25]. Here, we estimated the standardized beta per 1-SD increment for the Shannon index and richness. The analysis was performed with sequential adjustments: Model 1 was unadjusted (crude model); Model 2 was adjusted for age and TIV (main model); and Model 3 was adjusted for Model 2 plus BMI, physical activity, hypertension, smoking, and drinking (over-adjusted model). We tested the differences in between-person diversity (beta diversity) using principal coordinate analysis (PCoA) with permutational multivariate analysis of variance using Bray–Curtis distance matrices. Here, pseudo-F ratios were generated, and P values could be determined through permutations; a similar analysis was performed in an earlier study [25]. We performed linear discriminant analysis (LDA) in which the final output consisted of a list of microbiomes that were mostly discriminative at the family and genus levels and ranked according to the effect size with which they differentiated among groups. An LDA model was built with the class as the dependent variable and the remaining feature values, subclass, and subject values as independent variables (microbiome). This model is used to estimate the effect sizes, which were obtained by averaging the differences between class means (using unmodified feature values) and the differences between class means along the first linear discriminant axis, which equally weights feature variability and discriminatory power. The LDA score for each biomarker was obtained by computing the logarithm (base 10) of this value after being scaled in the interval, and, regardless of the absolute values of the LDA score, it ranked biomarker relevance. For robustness, LDA was additionally supported by bootstrapping (default 30-fold) and subsequent averaging. Raw taxonomic counts were transformed for analysis into log10[(RC/n) (x/N)+1], where RC is the total raw taxon count for a participant, n is the total count across all taxa for a participant, x is the total number of operational taxonomic units and participants, and N is the total number of participants. To account for spurious findings due to rare taxa, we restricted the analysis to taxa present in at least 50% of the participants.

Furthermore, we generated a heat map of associations between the microbiota genera and BVs (GMV and WMV) using multivariable-adjusted linear regression models. The direction of association is indicated by color, and FDR-adjusted P-values (q-values) are indicated by shading (blue, negative; red, positive). The gray matter and white matter were divided by TIV, and we performed three statistical models. Model 1 was unadjusted; Model 2 was adjusted for age and TIV; and Model 3 was adjusted for age, BMI, physical activity, hypertension, smoking, and drinking.

All analyses were performed using SAS version 9.4 (SAS Institute, Cary, NC, USA) and the R package maaslin. Taxonomic assignment was performed using QIIME2 (version 2021.2) software. Covariates with skewed distributions were log-transformed, and missing data were not included in the analyses.

## Results

Demographic and clinical characteristics of the study partcipants shown in Table 1.The mean age of the participants was 68.0 (SD, 8.0) years. Total cholesterol, hypertension, alcohol consumption, smoking history, and history of anti-hypertensive drug intake were remarkably high among the participants.

**Table 1:** Demographic and clinical characteristics of 623 men aged 40 to 79 years in the SESSA Study (2010-2014), Shiga, Japan.

Characteristics	SESSA Participants (n = 623)
Age (years)	68.0 ± 8.0
BMI (kg/m^2^)	23.3 ± 2.8
SBP (mm Hg)	132.0 ± 17.1
DBP (mm Hg)	77.1 ± 10.4
Total cholesterol (mg/dL)	202.3 ± 33.7
HDL-C (mg/dL)	59.8 ± 16.8
Triglyceride (mg/dL)	100.0 (71.0-140.0)
Physical activity, days/week	2.5 ± 2.6
Total brain volume (mL)	1082.4 ± 33.7
Gray matter (mL)	581.1 ± 49.4
White matter (mL)	501.3 ± 51.3
Hippocampus (mL)	7.94 ± 1.04
Total intracranial volume (mL)	1605.1 ± 109.6
Hypertension (yes)	351 (56.4)
Diabetes (yes)	129 (20.8)
Drinking alcohol	
Current	503 (80.8)
Past	31 (4.98)
Never	88 (14.1)
Smoking	
Current	116 (18.6)
Past	377 (60.6)
Never	129 (20.7)
Anti-lipid medication (yes)	146 (23.4)
Anti-diabetic medication (yes)	87 (13.10)
Antihypertensive medication (yes)	242 (38.9)

All values are presented as means ± SD for continuous variables except for triglycerides, white matter lesion volume [median, Interquartile ranges (IQR)], frequency, and percentages for categorical variables. The number of observations across categories may not add to the total number given because of missing data. BMI, body mass index; HDL-C, high-density lipoprotein cholesterol; LDL-C, low-density lipoprotein cholesterol; HR, HbA1c, hemoglobin A1c; SBP, systolic blood pressure; DBP, diastolic blood pressure. SESSA, Shiga Epidemiological Study of Subclinical Atherosclerosis

We performed multivariable adjusted linear regression to assess the association between gut microbial alpha diversity (Shannon index) and BVs (Table 2). In Model 1 (crude model), Shannon index was inversely associated with TBV (β = −2.74, CI= [−4.83, −0.66], P < 0.01) and hippocampal volume (β = −0.13, CI= [−0.21, −0.05], P < 0.01) but positively associated with GMV (β = 1.11, CI= [0.78–3.44], P < 0.01). After adjusting for age and TIV in Model 2 (main model), we found only GMV to be positively associated with Shannon index (β = 1.06, CI= [0.67–5.80], P = 0.041). In Model 3 (the over-adjusted model), which additionally adjusted for BMI, physical activity, smoking, drinking, and hypertension, this association was attenuated.

**Table 2:** Multivariable-adjusted associations between gut microbial alpha diversity (Shannon index) and brain volume measures in the SESSA Study (2010-2014).

	Total Brain Volume (mL)	White Matter (mL)	Gray Matter (mL)	Hippocampus (mL)
Shannon Index ^a^	**β *(95% CI)***	**β *(95% CI)***	**β *(95% CI)***	**β *(95% CI)***
Model 1	−2.74* (−4.83, −0.66)	−3.39 (−7.06, 0.26)	1.11* (0.78, 3.44)	−0.13* (−0.21, −0.05)
Model 2	1.48 (−1.37, 4.34)	2.63 (−1.78, 7.06)	1.06* (0.67, 5.80)	−0.03 (−0.12, 0.05)
Model 3	1.05 (−1.83, 3.95)	2.28 (−2.16, 6.73)	0.25 (−4.55,5.06)	−0.04 (−0.13, 0.04)
	Total Brain Volume (mL)	White Matter (mL)	Gray Matter (mL)	Hippocampus (mL)
Shannon Index ^a^	**β *(95% CI)***	**β *(95% CI)***	**β *(95% CI)***	**β *(95% CI)***
Model 1	−2.74* (−4.83, −0.66)	−3.39 (−7.06, 0.26)	1.11* (0.78, 3.44)	−0.13* (−0.21, −0.05)
Model 2	1.48 (−1.37, 4.34)	2.63 (−1.78, 7.06)	1.06* (0.67, 5.80)	−0.03 (−0.12, 0.05)
Model 3	1.05 (−1.83, 3.95)	2.28 (−2.16, 6.73)	0.25 (−4.55,5.06)	−0.04 (−0.13, 0.04)

Multivariable-adjusted linear regression models Model 1: unadjusted.

Model 2: adjusted for age and TIV

Model 3: Adjusted for age, TIV, BMI, physical activity, smoking, drinking, and hypertension.

^a^Associations are per standard deviation unit of genus-level diversity measures: Shannon index, means (SD) =5.70 (0.66). β, beta coefficient; SESSA, Shiga Epidemiological Study of Subclinical Atherosclerosis; TIV, total intracranial volume; mL, milliliters * P value <0.050.

**Fig 1:**
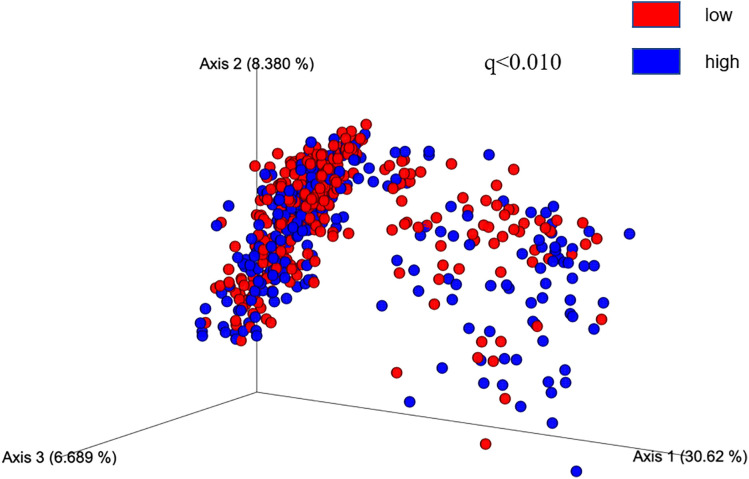
Beta diversity of gut microbiomes in GMV in SESSA Study (2010-2014). Legend: Weighted UniFrac Bray–Curtis distances measured by PCoA showed lower (blue) and higher (red) brain volumes formed distinct clusters in the case of WMV. We used the median value as the cutoff for defining higher and lower brain volumes (498.4 mL for WMV). PCoA axes explain at least 30% of variability in microbial similarity. Axis 1 explained 30.62% variance, axis 2 explained 8.38% variance, and axis 3 explained 6.7% variance.SESSA, Shiga Epidemiological Study of Subclinical Atherosclerosis; WMV, white matter volume; q is the FDR-adjusted P value (level of significance <0.05); PCoA,principal coordinate analysis.

**Fig 2:**
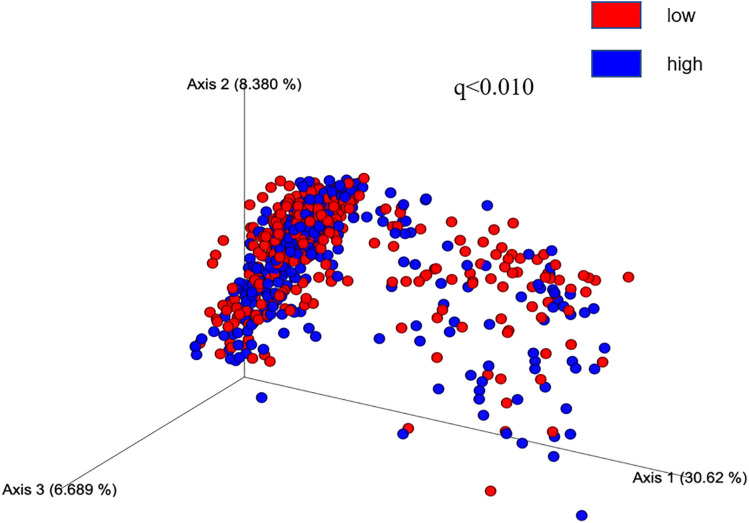
Beta diversity of gut microbiomes in WMV in the SESSA Study (2010-2014). Legend: Weighted UniFrac Bray–Curtis distances measured by PCoA showed lower (blue) and higher (red) brain volumes formed distinct clusters in the case of WMV. We used the median value as the cutoff for defining higher and lower brain volumes (498.4 mL for WMV). PCoA axes explain at least 30% of variability in microbial similarity.Axis 1 explained 30.62% variance, axis 2 explained 8.38% variance, and axis 3 explained 6.6% variance. SESSA, Shiga Epidemiological Study of Subclinical Atherosclerosis; WMV, white matter volume; q is the FDR-adjusted P value (level of significance <0.05);); PCoA,principal coordinate analysis.

Similarly, after performing a multivariable linear regression analysis to assess the association between gut microbial alpha diversity (richness) and BV, we found similar results (S1 Table). In the main model, adjusted for age and TIV, richness showed a positive association with TBV (β = 3.13, CI= [0.26–6.01], P = 0.032) and GMV (β = 4.37, CI= [0.30–9.49], P = 0.048); however, it was also attenuated in the over-adjusted model, which additionally adjusted for BMI, physical activity, hypertension, smoking, and drinking. After performing the PCoA analysis, the Bray–Curtis distance showed that the lower and higher groups formed distinct clusters in terms of GMV and WMV (FDR-adjusted P-value <0.01) when the median value for each volume was the cutoff (Figs 1 and 2). Each PCoA axis explained at least 30% of the variability in microbial similarity, suggesting variability among microbiomes in terms of different BVs.

**Fig 3:**
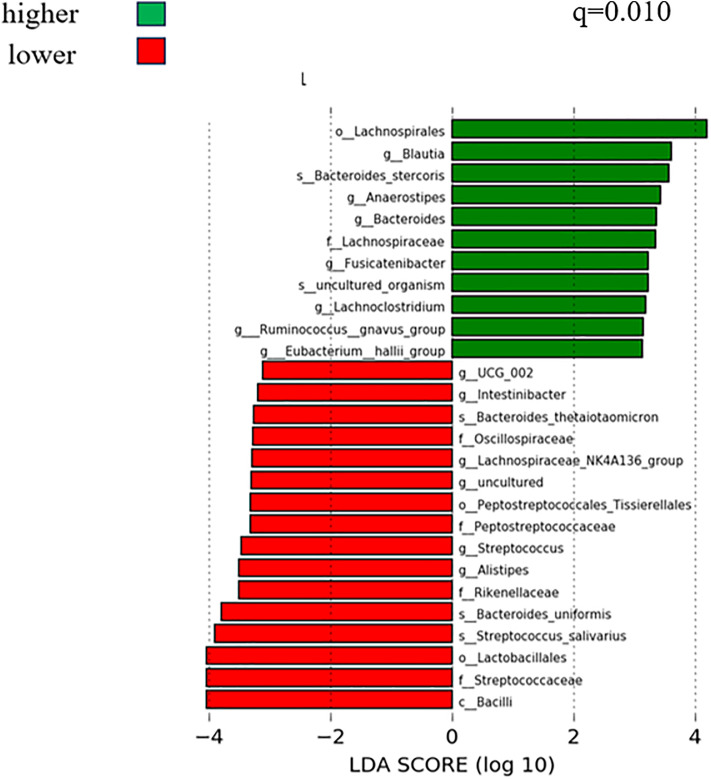
Linear discriminant analysis to identify taxa that were present in at least 50% of participants’ WMV in the SESSA Study (2010-2014). **Legend**: LDA showing microbiomes related to higher and lower WMV. Green indicates microbiomes associated with higher BVs, and red indicates microbiomes associated with lower BVs. q is the FDR-adjusted P value (level of significance <0.05); WMV, white matter volume; BVs, brain volumes; FDR, false discovery rate; PCoA, principal coordinate analysis; LDA, linear discriminant analysis; SESSA, Shiga Epidemiological Study of Subclinical Atherosclerosis.

**Fig 4:**
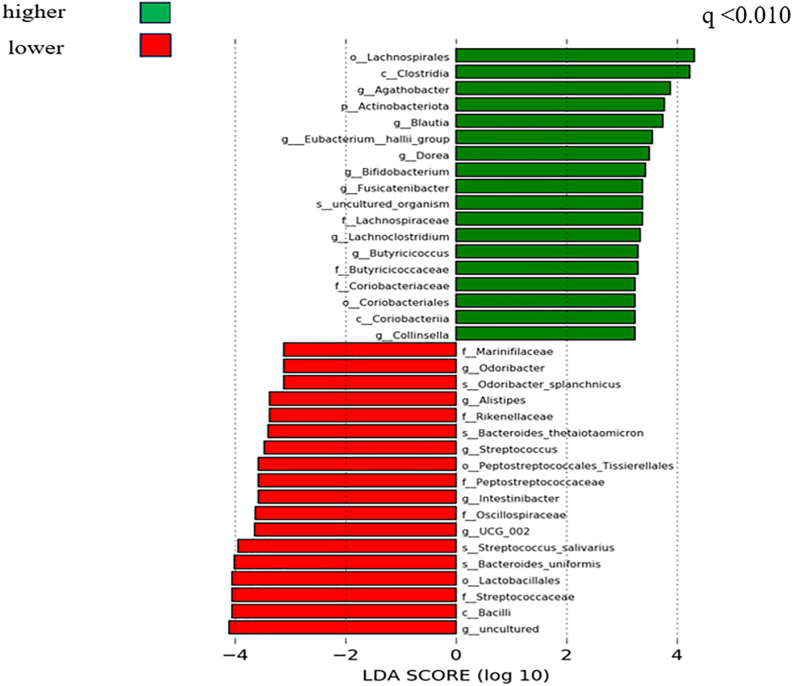
Linear discriminant analysis to identify taxa that were present in at least 50% of participants’ GMV in the SESSA Study (2010-2014). **Legend**: LDA (Linear Discriminant Analysis) shows microbiomes were related to higher and lower GMV. Green indicates microbiomes associated with higher BVs, and red indicates microbiomes associated with lower BVs. q is the FDR-adjusted P value (level of significance <0.05); GMV, gray matter volume; BVs, brain volumes; FDR, false discovery rate; PCoA, principal coordinate analysis; SESSA, Shiga Epidemiological Study of Subclinical Atherosclerosis.

However, as shown in S2 Fig, findings in the hippocampus (Bray–Curtis distance) were similar like Figs 1 and 2. As shown in Figs 3 and 4, we performed LDA to create a list of microbiomes and found that Lachnospiraceae, Lachnospirales, Blautia, Ruminococcus, and Lachnoclostridium were discriminative at different levels. We ranked them according to the effect size with which they differentiated between the higher and lower WMV and GMV using the median value as the cutoff. To account for spurious findings due to rare taxa, we restricted the analysis to taxa that were present in at least 50% of the participants and identified a total of 46 genera to be included in the analysis.

In S3 Fig, LDA showed the microbiomes related to higher and lower BVs in the hippocampus. Lachnospirales, Blautia, Ruminococcus, Fusicutenibacter, and Lachnoclostridium were associated with a higher hippocampal volume.

**Fig 5:**
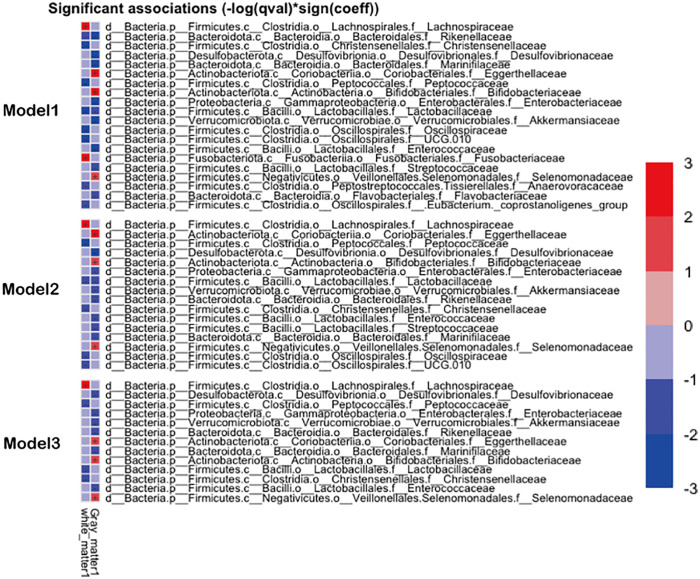
Multivariable adjusted linear regression analysis to identify taxa that were present in at least 50% of participants in the SESSA Study (2010-2014) in terms of GMV and WMV. Legend: Heat map of the associations between microbiomes and brain volumes (GMV and WMV) from multivariable-adjusted linear regression models. The direction of association is indicated by color (red, positive; blue, negative association; q, FDR-adjusted P values (<0.01). Model 1: unadjusted; Model 2: adjusted for age and total intracranial volume (TIV) Model 3: adjusted for age, physical activity, hypertension, smoking, and drinking. SESSA, Shiga Epidemiological Study of Subclinical Atherosclerosis; GMV, gray matter volume; WMV, white matter volume.

Furthermore, as shown in Fig 5, we created a heat map of the associations between the genera of microbiomes and BVs (GMV and WMV) using multivariable-adjusted linear regression models. In all models (Models 1–3), Lachnospiraceae was consistently positively associated with greater WMV; however, in Model 3, after adjusting for BMI, hypertension, physical activity, smoking, and drinking, Egarthellaceae, Bifidobacteraceae, and Selenomonadaceae showed a positive association with greater GMV.

As shown in S4 Fig, we created a heat map of associations between the genera of microbiomes and the hippocampus using multivariable-adjusted linear regression models. In all models (Models 1–3), Lachnospiraceae was consistently positively associated with a greater volume of the hippocampus.

## Discussion

To the best of our knowledge, this is the first study to report a correlation between the gut microbiome and BV in a general population globally. The main findings are as follows: (1) both the alpha diversity Shannon index and richness showed a positive correlation with the GMV and TBV after controlling for age and TIV, (2) the Lachnospiraceae bacteria group was consistently related to a high brain WMV, and Egarthellaceae, Bifidobacteraceae, and Selenomonadaceae showed an association with greater GMV.

The present study elucidated the gut microbiota in a regional cohort of 623 Japanese men. A previous study reported that the gut microbiota varies markedly among countries and living environments [26]. Therefore, the present results on the gut microbiota of 623 Japanese men are important because of the lack of data on the gut microbiome of Asians obtained from next-generation sequencing. Moreover, preventing unhealthy aging through personalized or subpopulation-level microbiome-associated interventions in healthy humans is crucial [12,13]; therefore, we focused on the relationship between the gut microbiome and BVs among the general population.

We report significant positive associations between gut microbiome alpha diversity and TBV and GMV after controlling for age and TIV. Regarding specific alpha diversity measures, GMV was significantly associated with the Shannon index (evenness and richness), whereas TBV and GMV were positively associated with species richness. However, after controlling for all the covariates, no significant association was observed between alpha diversity and BV. An earlier study conducted among individuals with depression reported that gut microbiome alpha diversity and hippocampal and nucleus accumbens volumes were correlated, after controlling for age and sex [27]. However other study conducted among patients with schizophrenia revealed both the alpha diversity evenness and Shannon indices showed a positive correlation with the GMV and ReHo indexes in several brain regions [11]; the difference in these results might be due to differences among the study participants. In our study, all participants were mostly healthy adults; however, earlier studies have discussed specific patient populations [11,27]. Increasing evidence suggests that gut alpha diversity is related to many age-associated changes, including immune system dysregulation and susceptibility to diseases [28,29]. The gut microbiota undergoes extensive changes throughout the lifespan, and age-related processes may influence gut microbiota diversity [30].

Regarding between-person (beta) diversity, our study revealed that the Bray–Curtis distance formed distinct clusters in terms of higher and lower GMV, WMV, and hippocampal volume. An earlier study reported a significant between-group difference in the Bray–Curtis distance between patients with schizophrenia patients and non-affected participants [11]. Similar findings were also reported among hypertensive and non-hypertensive groups in Coronary Artery Risk Development in Young Adults (CARDIA) study participants [25].

Earlier studies on patient populations have reported positive associations between several individual taxa and BVs. For example, in schizophrenia patients, the relative abundances of Ruminococcus, Roseburia, and Veillonella were significantly related to higher and lower BVs [11]. Another study found that Oxilabactor was related to GMV among elderly patients with depression [27]. However, in the current study, we did not find any such association between Oxilabactor and BVs. Since the present study participants were considered healthy, we found some groups of microbiota to be associated with higher and lower GMV and WMV, which was not reported in specific patient populations in earlier studies [9,11,27]. However, we found Lachnospiracae to be associated with greater GMV, WMV, and hippocampal volume after LDA, and the Lachnospiracae group was consistently associated with greater WMVs and hippocampal volumes in all three models in linear regression analysis. We identified Firmicutes at the class level, Clostridia at the order level, Lachnospirales at the family level, and Lachnospiraceae at the genus level. Of these, the relative abundance of the genus Lachnospiraceae demonstrated highly significant and strongly positive associations with WMV after controlling for age, BMI, physical activity, smoking, drinking, and hypertension. Owing to the cross-sectional study design, we were unable to establish causal links between individual taxa and BV, and it is unclear whether increased Lachnospiraceae contributes to the prevention of gray and white matter atrophy in the brain. Existing literature states that Lachnospiraceae is the most remarkable butyrate-producing species [31]. Short chain fatty acids (SCFAs), especially butyrate, have been reported to be the major source of nutrition for colonic epithelial cells [32–34].SCFA activity modulates the surrounding microbial environment and directly interacts with the host immune system [35]. Evidence from various studies suggests that Lachnospiraceae may play a role in supporting healthy functions, although certain genera and species within this family are found to be elevated in disease conditions [36]. Several diseases, such as metabolic syndrome, obesity, diabetes, liver diseases, inflammatory bowel disease, and chronic kidney diseases are all inflammatory conditions involving the Lachnospiraceae family or specific taxa of Lachnospiraceae [37–39] and, butyrate administration recovered memory function in a mouse model of Alzheimer’s disease [40]. A recent meta-analysis revealed differences in taxonomic composition and functional potential varied across studies among the patients with Alzheimer’s disease spectrum, and Lachnospiraceae were relatively reduced with aging among patients along the Alzheimer’s disease spectrum, leading to brain atrophy [41]; therefore, a high abundance of Lachnospiraceae may have a protective effect on brain health. However, further studies are strongly recommended for better understanding.

This study has several limitations that should be considered when interpreting the findings. First, its cross-sectional design restricts the ability to establish causal or temporal relationships between variables. Additionally, the cohort was composed exclusively of Japanese men, which may limit the generalizability of the results to other populations. While associations between microbiome diversity and brain volumes were identified, these were attenuated after adjusting for key confounders such as BMI, physical activity, and lifestyle factors, indicating the possibility of residual confounding. Reliance on 16S rRNA sequencing limits taxonomic resolution and functional insight into the microbiome’s metabolic output. Although several bacterial genera showed statistical associations with brain volumes, the clinical relevance and underlying biological mechanisms remain unclear, highlighting the need for future longitudinal and mechanistic studies. We could not include data related to diet and educational attainment in analysis owing to the lack of particular information.. Finally, these data were obtained once during the follow-up examination of the SESSA Study, and currently, we do not have any further follow-up data, to report on further assessment of BV alterations.

## Conclusion

In conclusion, our findings support an association between gut microbiome diversity and BV in middle-aged to older Japanese men. Microbial diversity was positively associated with BV. Several specific genera were significantly associated with BV after adjusting for potential confounders. This study generated a hypothesis that controlling gut flora might affect age-related brain volume alternation. Further longitudinal studies on different populations are required to better understand the observed associations.

## Supporting information

S1 Table Multivariable-adjusted associations between gut microbial alpha diversity (richness) and brain volume measures in the SESSA Study (2010–2014). Legend: Multivariable adjusted linear regression models; Model 1: unadjusted Model 2: adjusted for age and TIV, Model 3: Adjusted for age, BMI, physical activity, smoking, drinking, and hypertension. Associations are presented per standard deviation unit of genus-level diversity measures: richness (log) means (SD) =6.78 (0.27). β, beta coefficient; BMI, body mass index; SESSA, Shiga Epidemiological Study of Subclinical Atherosclerosis; TIV, total intracranial volume.
(PDF)

S1 Fig Partcipants Recruitment Flow Chart of the study. Legend: The flow diagram shows the participant inclusion steps. SESSA, Shiga Epidemiological Study of Subclinical Atherosclerosis; MRI, magnetic resonance imaging; rRNA, ribosomal ribonucleic acid.
(PDF)

S2 Fig Beta diversity of gut microbiomes in Hippocampus in the SESSA Study (2010–2014). Legend: Weighted UniFrac Bray–Curtis distances measured by principal coordinate analysis showed lower brain volumes (blue) and higher brain volumes (red) in the hippocampus formed distinct clusters. We used median value as cutoff for defining higher and lower brain volumes. PCoA axes explain at least 30% of variability in microbial similarity. Axis 1 explained 30.62% variance, axis 2 explained 8.38% variance, and axis 3 explained 6.6% variance. SESSA, Shiga Epidemiological Study of Subclinical Atherosclerosis; q is the FDR-adjusted P value (level of significance <0.05).
(PDF)

S3 Fig Linear discriminant analysis (LDA) shows microbiomes related to higher and lower brain volumes in the hippocampus. Legend: LDA shows microbiomes related to higher and lower hippocampal brain volumes. q is the FDR-adjusted P value (level of significance<0.05); FDR, False Discovery Rate; PCoA, Principal Coordinate Analysis, SESSA, Shiga Epidemiological Study of Subclinical Atherosclerosis.
(PDF)

S4 Fig Multivariable adjusted linear regression analysis to identify taxa that were present in the hippocampus in at least 50% of participants in SESSA Study (2010–2014). Legend: Heat map of associations between microbiomes and brain volume (hippocampus) from multivariable-adjusted linear regression models. The direction of association is indicated by color (red, positive; blue, negative association; q, FDR-adjusted P values (<0.01).Model 1: unadjusted; Model 2: adjusted for age and total intracranial volume; Model 3: adjusted for age, total intracranial volume, body mass index, physical activity, hypertension, smoking, and drinking. SESSA: Shiga Epidemiological Study of Subclinical Atherosclerosis.
(PDF)
