# Supplementary material for: Relationship between Gut microbiome and brain volumes among Japanese Men
Source: PLoS One. 2025 Oct 7;20(10):e0333612. doi: 10.1371/journal.pone.0333612 (PMC12503305; doi:10.1371/journal.pone.0333612)
Supplement: S1 Table — Legend: Multivariable adjusted linear regression models; Model 1: unadjusted Model 2: adjusted for age and TIV, Model 3: Adjusted for age, BMI, physical activity, smoking, drinking, and hypertension. Associations are presented per standard deviation unit of genus-level diversity measures: richness (log) means (SD) =6.78 (0.27). β, beta coefficient; BMI, body mass index; SESSA, Shiga Epidemiological Study of Subclinical Atherosclerosis; TIV, total intracranial volume. (PDF) [file pone.0333612.s001.pdf]

Supplementary Table S1. Multivariable-adjusted associations between gut microbial alpha diversity (richness) and brain volume measures in the SESSA Study (2010-2014)

	Total Brain Volume (mL)	White Matter (mL)	Gray Matter (mL)	Hippocampus (mL)
Richness	β *(95% CI)*	β *(95% CI)*	β *(95% CI)*	β *(95% CI)*
Model 1	-1.18 (-3.28, 0.90)	-1.86 (-5.53, 1.81)	-1.81 (-5.50, 1.87)	-0.06 (-0.15, 0.09)
Model 2	3.13* (0.26, 6.01)	3.38 (-1.06, 7.84)	4.37* (0.30, 9.49)	0.03 (-0.05, 0.12)
Model 3	2.81 (-0.09, 5.72)	3.08 (-1.39, 7.56)	4.16 (-0.66, 8.99)	0.02 (-0.07, 0.11)

Multivariable adjusted linear regression models

Model 1: unadjusted

Model 2: adjusted for age and TIV

Model 3: Adjusted for age, BMI, physical activity, smoking, drinking, and hypertension. Associations are presented per standard deviation unit of genus-level diversity measures: richness (log) means (SD) =6.78 (0.27). β, beta coefficient; BMI, body mass index; SESSA, Shiga Epidemiological Study of Subclinical Atherosclerosis; TIV, total intracranial volume.
