# Supplementary material for: Relationship between Gut microbiome and brain volumes among Japanese Men
Source: PLoS One. 2025 Oct 7;20(10):e0333612. doi: 10.1371/journal.pone.0333612 (PMC12503305; doi:10.1371/journal.pone.0333612)
Supplement: S1 Fig — Legend: The flow diagram shows the participant inclusion steps. SESSA, Shiga Epidemiological Study of Subclinical Atherosclerosis; MRI, magnetic resonance imaging; rRNA, ribosomal ribonucleic acid. (PDF) [file pone.0333612.s002.pdf]

Supplementary Fig S1.

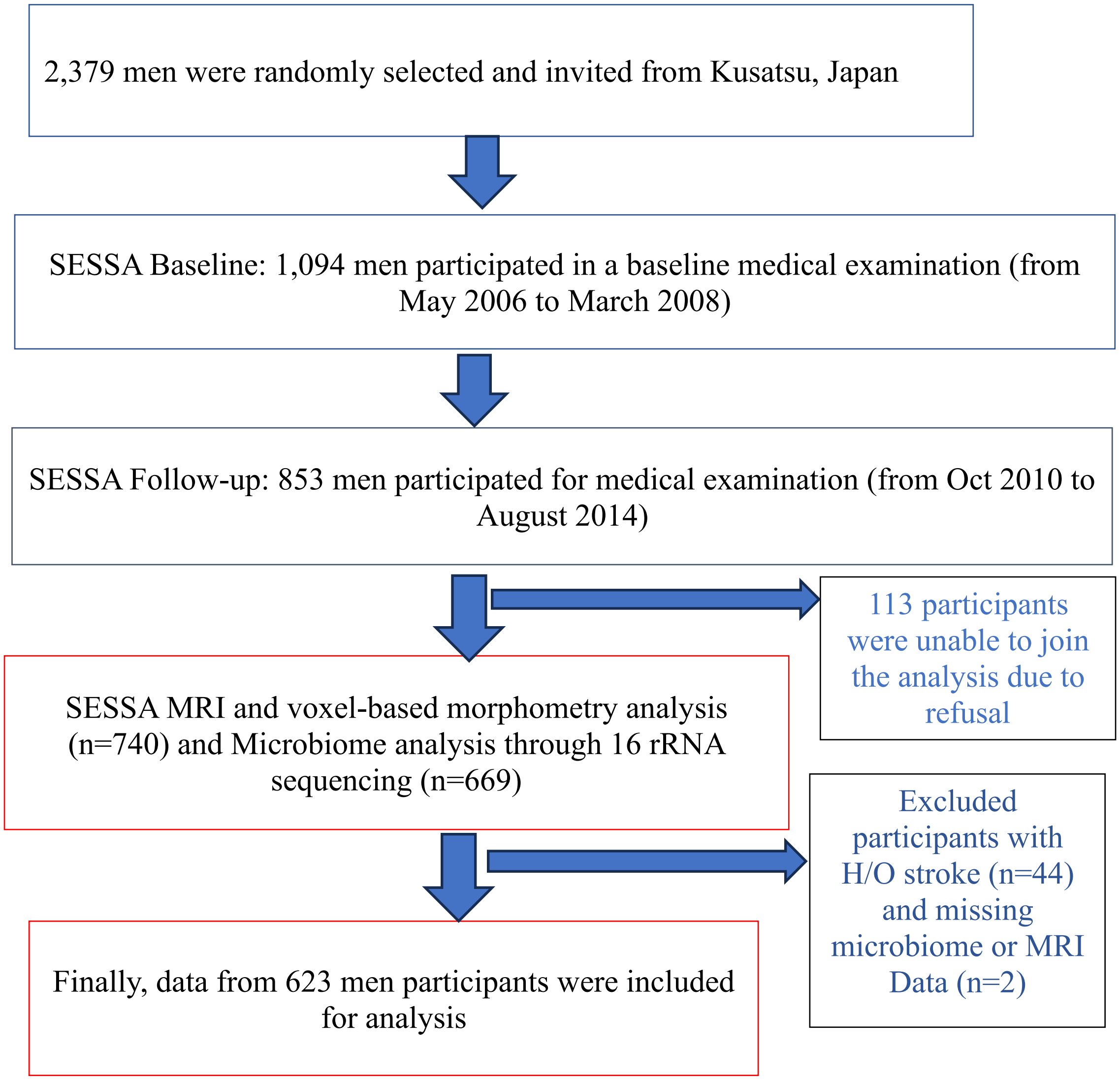

Figure legend: The flow diagram shows the participant inclusion steps. SESSA, Shiga Epidemiological Study of Subclinical Atherosclerosis; MRI, magnetic resonance imaging; rRNA, ribosomal ribonucleic acid.
