# Supplementary material for: Relationship between Gut microbiome and brain volumes among Japanese Men
Source: PLoS One. 2025 Oct 7;20(10):e0333612. doi: 10.1371/journal.pone.0333612 (PMC12503305; doi:10.1371/journal.pone.0333612)
Supplement: S2 Fig — Legend: Weighted UniFrac Bray–Curtis distances measured by principal coordinate analysis showed lower brain volumes (blue) and higher brain volumes (red) in the hippocampus formed distinct clusters. We used median value as cutoff for defining higher and lower brain volumes. PCoA axes explain at least 30% of variability in microbial similarity. Axis 1 explained 30.62% variance, axis 2 explained 8.38% variance, and axis 3 explained 6.6% variance. SESSA, Shiga Epidemiological Study of Subclinical Atherosclerosis; q is the FDR-adjusted P value (level of significance <0.05). (PDF) [file pone.0333612.s003.pdf]

Supplementary Figure S2.

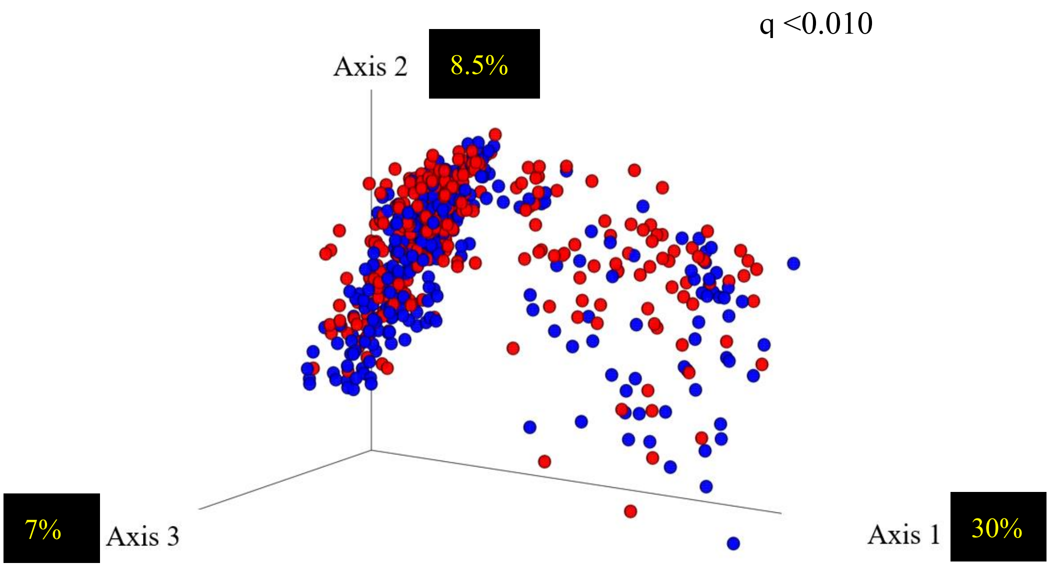

Figure legend: Weighted UniFrac Bray–Curtis distances measured by principal coordinate analysis showed lower brain volumes (blue) and higher brain volumes (red) in the hippocampus formed distinct clusters. We used median value as cutoff for defining higher and lower brain volumes. PCoA axes explain at least 30% of variability in microbial similarity. Axis 1 explained 30.62 % variance, axis 2 explained 8.38% variance, and axis 3 explained 6.6% variance. SESSA, Shiga Epidemiological Study of Subclinical Atherosclerosis; q is the FDR-adjusted P value (level of significance <0.05).
