# Supplementary material for: Relationship between Gut microbiome and brain volumes among Japanese Men
Source: PLoS One. 2025 Oct 7;20(10):e0333612. doi: 10.1371/journal.pone.0333612 (PMC12503305; doi:10.1371/journal.pone.0333612)
Supplement: S3 Fig — Legend: LDA shows microbiomes related to higher and lower hippocampal brain volumes. q is the FDR-adjusted P value (level of significance<0.05); FDR, False Discovery Rate; PCoA, Principal Coordinate Analysis, SESSA, Shiga Epidemiological Study of Subclinical Atherosclerosis. (PDF) [file pone.0333612.s004.pdf]

Supplementary Fig. S3. Linear discriminant analysis (LDA) shows microbiomes related to higher and lower brain volumes in the hippocampus.

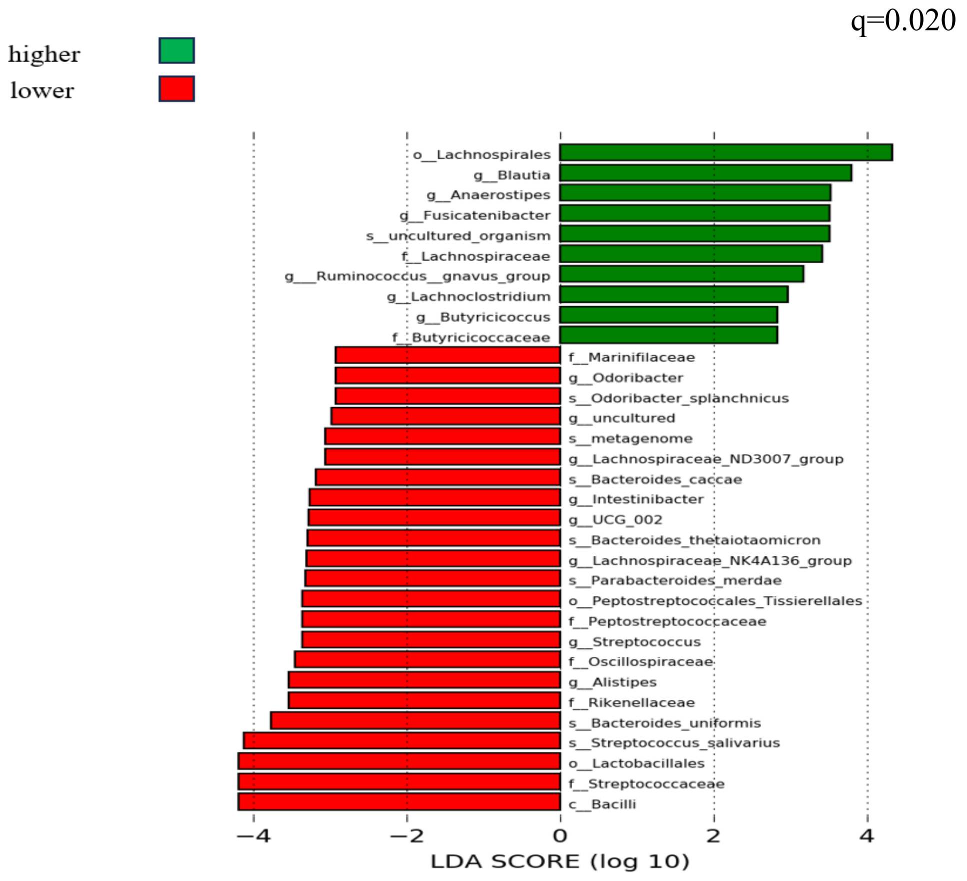

Legend: LDA shows microbiomes related to higher and lower hippocampal brain volumes. q is the FDR-adjusted P value (level of significance <0.05); FDR, False Discovery Rate; PCoA, Principal Coordinate Analysis, SESSA, Shiga Epidemiological Study of Subclinical Atherosclerosis.
