# Supplementary material for: Relationship between Gut microbiome and brain volumes among Japanese Men
Source: PLoS One. 2025 Oct 7;20(10):e0333612. doi: 10.1371/journal.pone.0333612 (PMC12503305; doi:10.1371/journal.pone.0333612)
Supplement: S4 Fig — Legend: Heat map of associations between microbiomes and brain volume (hippocampus) from multivariable-adjusted linear regression models. The direction of association is indicated by color (red, positive; blue, negative association; q, FDR-adjusted P values (<0.01).Model 1: unadjusted; Model 2: adjusted for age and total intracranial volume; Model 3: adjusted for age, total intracranial volume, body mass index, physical activity, hypertension, smoking, and drinking. SESSA: Shiga Epidemiological Study of Subclinical Atherosclerosis. (PDF) [file pone.0333612.s005.pdf]

**Supplementary Fig S4.** Multivariable adjusted linear regression analysis to identify taxa that were present in the hippocampus in at least 50% of participants in SESSA Study (2010-2014)

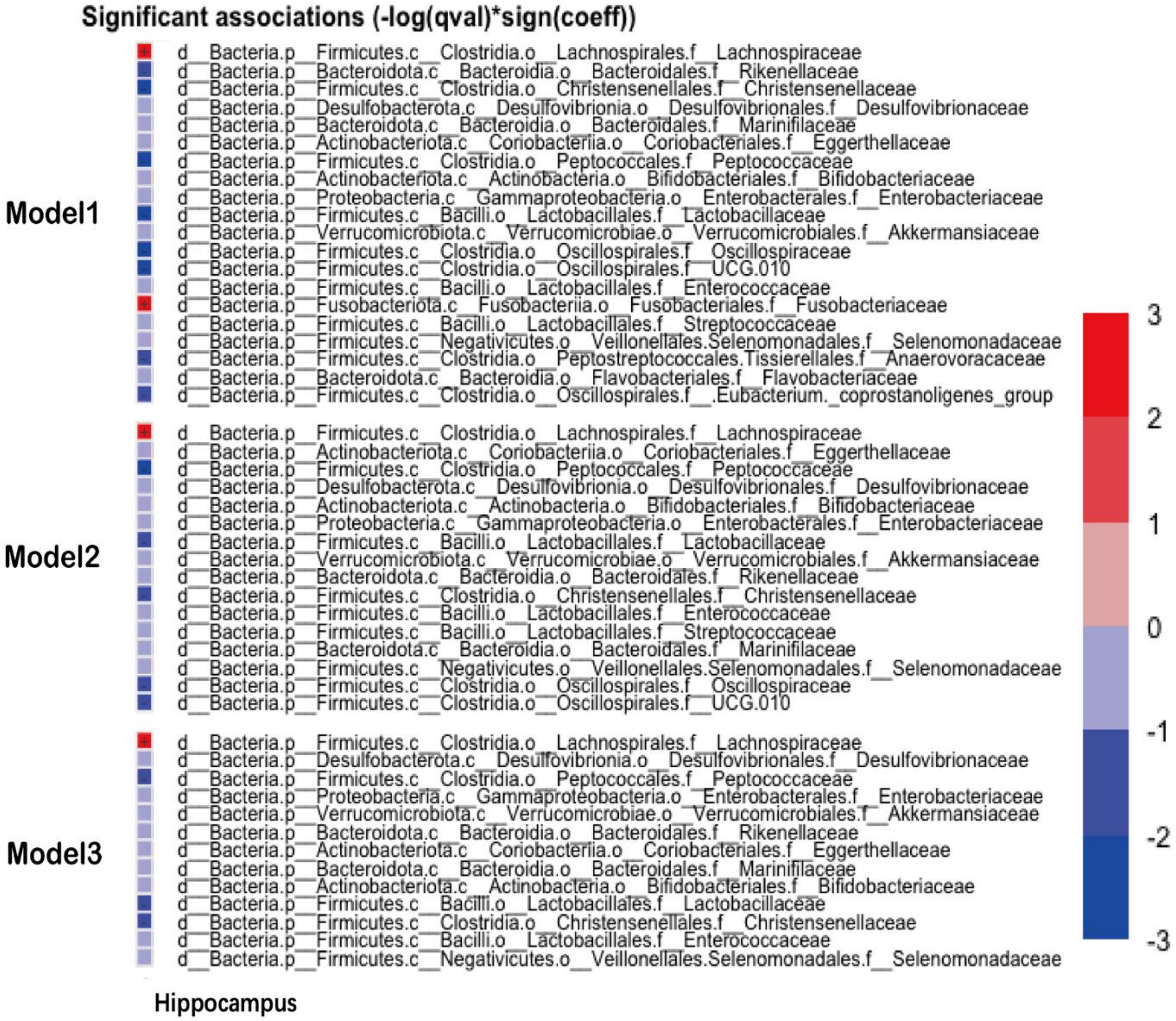


Heat map of associations between microbiomes and brain volume (hippocampus) from multivariable-adjusted linear regression models. The direction of association is indicated by color (red, positive; blue, negative association; q, FDR-adjusted P values (<0.01). Model 1: unadjusted; Model 2: adjusted for age and total intracranial volume; Model 3: adjusted for age, total intracranial volume, body mass index, physical activity, hypertension, smoking, and drinking. SESSA: Shiga Epidemiological Study of Subclinical Atherosclerosis.
